# DNA methylation profiling in mummified human remains from the eighteenth-century

**DOI:** 10.1038/s41598-021-95021-7

**Published:** 2021-07-29

**Authors:** Marco Schmidt, Frank Maixner, Gerhard Hotz, Ildikó Pap, Ildikó Szikossy, György Pálfi, Albert Zink, Wolfgang Wagner

**Affiliations:** 1 Helmholtz-Institute for Biomedical Engineering, Stem Cell Biology and Cellular Engineering, RWTH Aachen University Medical School, Pauwelsstrasse 20, 52074 Aachen, Germany; 2 Institute for Mummy Studies, Eurac Research, Viale Druso, 1, 39100 Bolzano, Italy; 3 Anthropological Collection, Natural History Museum of Basel, 4051 Basel, Switzerland; 4 Integrative Prehistory and Archaeological Science (IPAS), University of Basel, 4051 Basel, Switzerland; 5 Department of Biological Anthropology, Faculty of Science and Informatics, University of Szeged, 6726 Szeged, Hungary; 6 Department of Anthropology, Hungarian Natural History Museum, 1083 Budapest, Hungary; 7 Department of Biological Anthropology, Eötvös University, 1117 Budapest, Hungary

**Keywords:** Bioinformatics, Epigenetics analysis, Cells, DNA methylation, Palaeoecology

## Abstract

Reconstruction of ancient epigenomes by DNA methylation (DNAm) can shed light into the composition of cell types, disease states, and age at death. However, such analysis is hampered by impaired DNA quality and little is known how decomposition affects DNAm. In this study, we determined if EPIC Illumina BeadChip technology is applicable for specimens from mummies of the eighteenth century CE. Overall, the signal intensity on the microarray was extremely low, but for one of two samples we were able to detect characteristic DNAm signals in a subset of CG dinucleotides (CpGs), which were selected with a stringent processing pipeline. Using only these CpGs we could train epigenetic signatures with reference DNAm profiles of multiple tissues and our predictions matched the fact that the specimen was lung tissue from a 28-year-old woman. Thus, we provide proof of principle that Illumina BeadChips are applicable for DNAm profiling in ancient samples.

## Introduction

Under favorable conditions not only the DNA sequence, but also epigenetic marks can be preserved for thousands of years^1^. DNA methylation (DNAm) is an epigenetic mark, which is dynamically regulated during cellular differentiation^2^. Therefore, it is possible to estimate the cellular composition of a given sample based on deconvolution of DNAm patterns^3,4^. A multitude of diseases, such as leukemia or other malignancies, are reflected in characteristic DNAm profiles^5^, and this may support pathological analysis of human remains. Furthermore, so called “epigenetic clocks” enable estimation of donor age with relatively high precision^6–8^. Thus, the DNAm pattern can elucidate important parameters for paleogenomic and anthropological investigations.

However, epigenetic analysis of ancient samples is technically extremely challenging^1^. The content of endogenous human DNA in such samples is usually low due to DNA decomposition into ultrashort fragments and high abundance of microbial background DNA. Additionally, post-mortem base modifications occur particularly at the 5′-end of DNA fragments and thereby impact sequencing analysis^1,9^. Notably, cytosine residues of methylated CpGs are deaminated into thymidine, whereas those of unmethylated CpGs are converted into uracil. This feature was successfully exploited to estimate DNAm levels in high-throughput sequencing data of Neanderthal remains^10,11^. Yet, this method requires a very high sequencing depth, and it may not be applicable to more recent remains with fewer post most-mortem base modifications. Therefore, in this explorative study, we analyzed if DNAm profiling in human remains of the eighteenth century would also be feasible with Illumina BeadChip microarray technology^12^.

## Results

### DNA sequencing reveals C to T conversions

**Figure 1:**
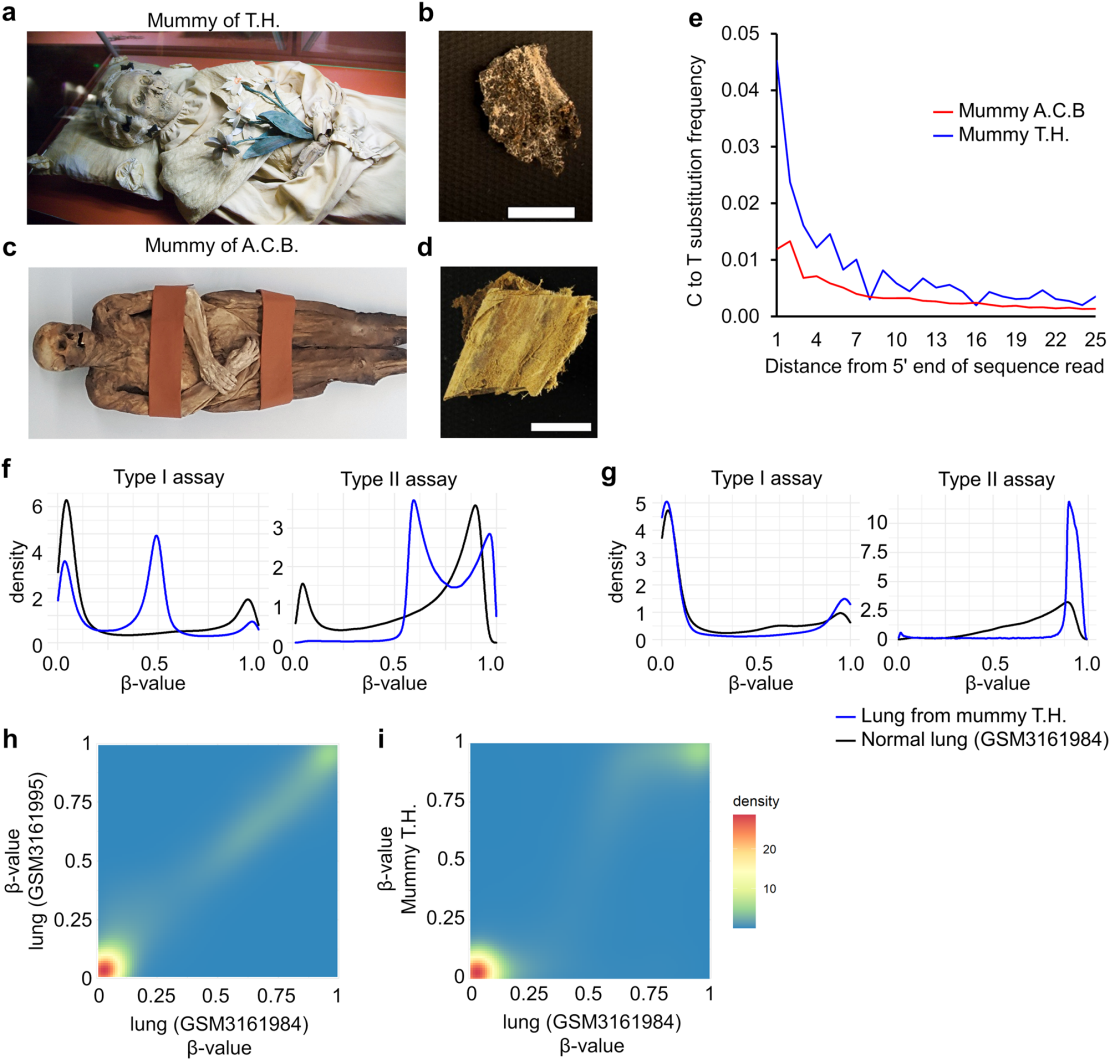
DNA methylation analysis in human remains. (**a**) Mummified human remains and (**b**) lung tissue sample of Terézia Hausmann (1769–1797); and (**c**) the remains and (**d**) gut tissue specimen of Anna Catharina Bischoff (A.C.B; 1719–1787); Size bar = 1 cm. (**e**) Frequency of specific base substitutions of cytosines into thymidine at the 5′-ends of sequencing reads. Both DNA samples display increased frequencies of C to T substitutions close to the ends of DNA fragments, which is characteristic of ancient DNA. (**f**) Density plots of DNAm levels of the T.H. sample (β-value) across all Type I and Type II assays represented by the Illumina EPIC BeadChip (after normalization with ssNoob). (**g**) Density plots of 23,875 CpGs in Type I assays and 42,161 CpGs in Type II assays that passed the quality filter criteria (SeSAMe: P < 0.01). (**h**) 2-D density plots comparing DNAm levels in the same 23,875 filtered Type I assays between two present day lung tissue samples (Pearson correlation = 0.98); and (**i**) between a present-day lung-tissue sample and the profile of the specimen of T.H. (Pearson correlation = 0.94, CpGs on allosomes and SNPs were removed).

We were given access to two samples from mummified human remains dating to the eighteenth century: (1) a lung tissue sample of Terézia Hausmann (T.H.; 1769–1797), who was suffering from tuberculosis and was discovered during reconstruction works in the Dominican church of Vác, Hungary (Fig. 1a,b)^13,14^, and (2) a gut tissue specimen of the corpse of Anna Catharina Bischoff (A.C.B.; 1719–1787) who was excavated in the Barfüsserkirche in Basel, Switzerland (Fig. 1c,d)^15,16^. The concentration of the isolated DNA was very low (16 ng/µl for A.C.B. and 11 ng/µl for T.H.) and shotgun sequencing of isolated DNA revealed that particularly in the sample from A.C.B. the read length was very short, which might indicate higher fragmentation (Supplemental Table S1, Supplemental Fig. S1a). Furthermore, taxonomic analysis of the sequencing reads demonstrated that the human endogenous DNA content was only 0.97% (A.C.B.) and 0.65% (T.H.) (Supplemental Fig. S1b). The frequency of C to T conversions at the 5´-end of sequencing reads was 0.012 and 0.045, respectively (Fig. 1e).

### DNA methylation profiles of ancient DNA require strict probe detection filtering

Genomic DNA was then bisulfite converted and analyzed on the EPIC Illumina BeadChip microarrays, which can address more than 850,000 CpGs by two different probe designs: type I assays consist of two bead types per CpG locus (one for unmethylated and one for methylated sequences), whereas type II assays have only one bead type that incorporates different fluorescently labeled nucleotides depending on the methylation status^12^. Notably, only approximately 320 ng and 220 ng of total DNA were hybridized, which only corresponds to about 3.1 ng and 1.4 ng of human DNA in the sample of A.C.B and T.H., respectively. This amount is well below the recommended amount of 250 ng and also less than in other studies that successfully tested dilutions with 50 ng^4^. Thus, the initial analysis revealed overall extremely low signal intensities. The signals were slightly higher for the mummy of T.H. than for A.C.B., despite the even lower fraction of human DNA (Supplemental Fig. S2a). Quality control probes for sample preparation steps also revealed that especially the sample of A.C.B. failed most of the quality thresholds (Supplemental Table S2). Furthermore, density plots for DNAm levels (β-values) did not show the typical bimodal distribution of methylated and unmethylated CpGs (Supplemental Fig. S2b). Yet, we observed small peaks at low and high DNAm levels for the T.H. sample, particularly for type I assays, indicating that a subset of CpGs might provide useful methylation signals (Fig. 1f). To filter for such CpGs, we used the SeSAMe package^17^ to select 23,875 CpGs of the type I assay with the lowest detection P-values (P < 0.01; Fig. 1g). When we compared DNAm levels in this subset of CpGs, we observed a clear correlation between DNAm levels of T.H. and normal lung-tissue (Fig. 1h,i). However, despite various approaches for normalization and filtering, we could not detect such an association in the DNAm measurement of A.C.B. (Supplemental Fig. S2c–e). Further analysis of DNAm patterns was therefore only performed for the T.H. specimen.

### Tissue-specific DNA methylation patterns in ancient DNA

**Figure 2:**
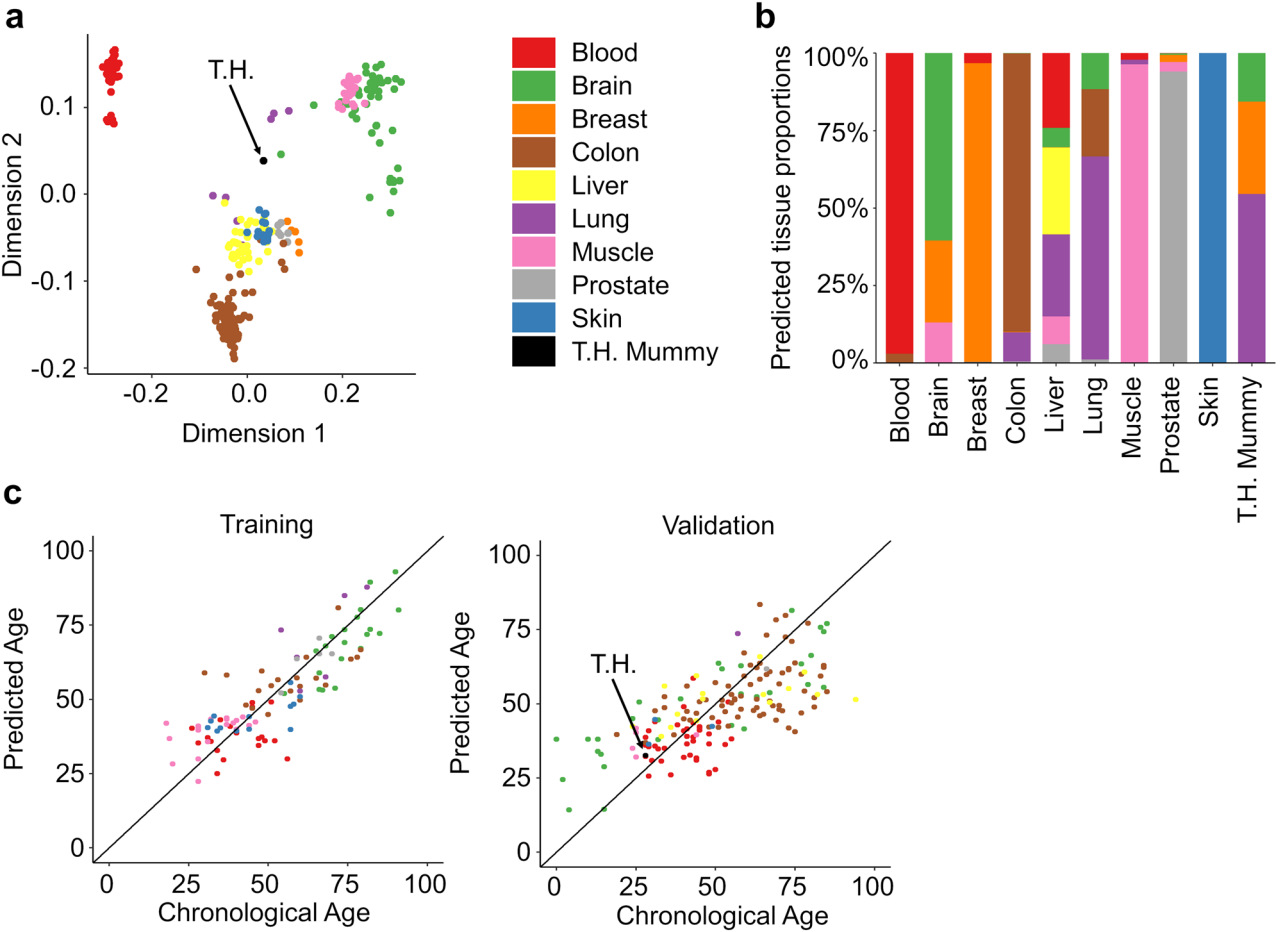
Epigenetic classification of the DNA methylation profile. For the subsequent DNAm classification of the sample from the mummy of T.H. we focused exclusively on 22,778 CpGs that passed the filter criteria. (**a**) Multidimensional scaling (MDS) plot of the top 100 most variable CpGs in 301 Illumina EPIC BeadChip profiles of nine different tissues and the mummy sample. (**b**) Tissue specific DNAm signatures were trained for the filtered CpGs (5 CpGs per tissue) and used for a deconvolution algorithm. The tissue-predictions are exemplary depicted for samples of the validation set and for the T.H. sample. (**c**) Age predictors were trained for 7 CpGs of the Horvath aging clock^7^ that were comprised in the filtered CpGs. Age-predictions correlated with chronological age in the training set (R^2^ = 0.71) and validation set (R^2^ = 0.43; two outliers are not depicted).

To estimate tissue-specific DNAm patterns, we focused only on the subset of detected CpGs (22,778 after removing CpGs that are associated with X and Y chromosome or single nucleotide polymorphisms [SNPs]) to facilitate better comparison with other datasets. As a reference, we compiled 301 public Illumina EPIC BeadChip datasets from nine different tissues (Supplemental Tables S3, S4). Multidimensional scaling (MDS) of DNAm with the 100 most variable CpGs revealed that the lung tissue sample of the mummy of T.H. clustered relatively close to other lung-tissue samples (Fig. 2a). To further estimate the cellular composition, we used a previously published reference methylation atlas of 25 human tissues and cell types, which utilizes 7890 CpGs for deconvolution of cell types^4^. However, only 578 of these CpGs were comprised in our subset. Despite this limitation, most of the tissues in our dataset collection could be correctly assigned to the corresponding tissue. However, this did not work reliably for lung tissue samples and for the lung specimen from T.H., possibly due to the small number of remaining CpGs in the signatures and due to the fact that the lung-specific CpGs were only selected with lung epithelial cells and not whole tissue (Supplemental Fig. S3).

Alternatively, we trained a new tissue deconvolution model that only utilizes the detected CpGs. To this end, we split the DNAm dataset collection into a training and validation set (Supplemental Table S4). For each of the nine tissues, we selected the top ranked candidate CpGs with tenfold cross-validation as described in our previous work^3^ (Supplemental Fig. S4). The mean DNAm levels in the nine tissues of the training dataset were then used as reference matrix for a non-negative least squares (NNLS) deconvolution algorithm. Overall, the predicted tissues corresponded to the real tissues in both the training and the validation set (Supplemental Fig. S5). Using this predictor, the normal lung tissue was merely predicted as lung tissue and very similar results were observed for the tissue sample of T.H., indicating that the DNAm profile still reflects the tissue of origin (Fig. 2b). For sex-analysis with DNAm profiles the conventional methods (R packages minfi, Ewastools, sEst, and watermelon) were not reliably applicable with the reduced set of detected CpGs. However, the percentage of detected CpGs was higher on the X than on the Y chromosome, which is in line with female samples (Supplemental Fig. S6).

### Epigenetic age predictions

Subsequently, we wanted to analyze if the DNAm was still indicative for age at death. To this end, we used a multi-tissue age predictor described by Horvath^7^. Only seven CpGs of this 353 CpGs epigenetic clock were within the detected 22,778 CpGs. Therefore, we have retrained an epigenetic age predictor based on these seven CpGs (Supplemental Table S5) and age-predictions with this reduced aging signature correlated with the chronological age for samples of the training (R^2^ = 0.71) and validation set (R^2^ = 0.43). Notably, the sample of T.H. was predicted to be 33 years old—and thus very close to documented age of 28 years (Fig. 2c).

## Discussion

Our exploratory study demonstrates that it is possible to address DNAm in human remains from the eighteenth century with Illumina BeadChip microarrays. At first sight, the samples failed the quality control step and would have normally been excluded from further analysis. It was necessary to adjust processing and the sample-specific analysis pipeline to the very low signal intensities. We chose ssNoob normalization (minfi)^18^ together with detection P-value filtering based on the SeSAMe package^17^. This provided a subset of CpGs with similar DNAm levels as observed in freshly isolated tissue. It is still unclear, why this was not possible for the mummy of A.C.B., albeit the detected fraction of human DNA was higher and C to T substitution frequency was lower. This might be partly attributed to higher fragmentation as indicated by the shorter read length in the shotgun sequencing results. The corpse of A.C.B. revealed very high levels of mercury sulfide, possibly for syphilis treatment, and it is conceivable that such conditions impact on DNAm measurements^19^.

In an elegant study, Pedersen et al. utilized the CpG to TpG substitutions at the start of sequencing reads to estimate DNAm levels in a 4000-year-old Paleo-Inuit sample^20^. In comparison to public Illumina 450 k BeadChip profiles, they could demonstrate that the estimated DNAm levels clustered with patterns of hair follicle—corresponding to the tissue used for DNA extraction^20^. Yet, this approach to detect cytosine methylation depends on the extend of post-mortem deamination rates and ultimately on the sequencing depth. In comparison, our method is relatively cost effective and facilitates a more direct comparison with other DNAm profiles that were generated on the same Illumina BeadChip platform. On the other hand, the bioinformatics requirement to train predictors specifically for a small subset of CpGs should not be underestimated. The low number of remaining CpGs reduces applicability. Our age-predictions were based on only seven out of 353 CpGs of the Horvath aging clock^7^, which we chose because it was trained for multiple different tissues. However, with a larger reference dataset available it might be advantageous to use all CpGs that passed the filter criteria and to establish an independent multi-tissue age-predictor for a given specimen. Furthermore, the impact of postmortem deamination needs to be considered, albeit the percentage is relatively low and enriched at the 5′ ends. It is yet unclear if the lower signal intensity is only a result of the low amount of DNA or rather to the low fraction of human DNA. It might be possible to use hybrid capture to enrich the entire human genome, therefore reducing the contamination of “foreign DNA”. Alternatively hybrid capture could even be used with a relatively small panel of regions that are important for tissue deconvolution and/or epigenetic age estimation^21,22^.

The findings of this study may also be relevant for forensics, since only few studies have analyzed DNAm changes in corpses at different stages of decomposition. We have recently described that age-associated DNAm changes in *PDE4C* that were analyzed in buccal swabs at a post mortem interval of 1 to 42 days were hardly affected by early decomposition^23^. Here we show that conventional microarray-based methods for DNAm profiling may even be applicable in mummified corpses after centuries. Recently, there is much progress in epigenetic biomarker development for lifestyle habits (e.g. smoking^24^ or diseases (e.g. malignant diseases^25^). Thus, DNAm analysis in ancient corpse might also shed additional insight into habits and prevalence of diseases in former days. However, the broad applicability is limited, since we were only successful for one of two samples. It will be necessary to further validate this approach with larger sample collections of multiple different mummies to better understand the relevant parameters and reliability of the results. Furthermore, it will be interesting to determine if this method is even applicable to much older remains.

## Methods

### Sample collection

We analyzed lung tissue specimens from the mummified human remains of Terézia Hausmann who lived in Vác, Hungary (Stored at the Hungarian Natural History Museum, Body 68, Inventory number: 2009.19.68; EURAC ID: 2367)^26^. A total of 265 well documented mummified individuals were discovered during reconstruction works in the Dominican church of Vác, Hungary, between 1994 and 1995. Constant temperatures (8–11 °C) in the crypts in combination with continuous ventilation led to a natural mummification of the buried human bodies in their coffins. The corpse of Anna Catharina Bischoff was discovered during reconstruction works inside the Barfüsserkirche in Basel, Switzerland, in 1975 in one of the excavated coffins. The mummy was identified in a multidisciplinary research project^15^ and during a sampling campaign in 2016 gut tissue material has been taken (EURAC ID: 2132). This study was conducted with approval of the museum collections and based on the International Council of Museums code of ethics (ICOM 2017) with regard to storage, display, and study of human remains. The study team carefully considered ethical issues and the appropriateness of the research involving human mummies, as human remains have to be considered not as ‘objects’ but as the remains of once-living people^27^. Several tissues of the mummies were initially probed, but for this exemplary analysis we simply used available tissue specimen. Lung tissue of T.H. was extensively probed for the tuberculosis study^26^.

### Extraction of DNA, library preparation, and sequencing

Sample preparation and DNA extraction was performed in a dedicated pre-PCR area with protective clothing, UV-light exposure of the equipment, bleach sterilization of surfaces and filtered pipette tips at the ancient DNA laboratory of the EURAC Institute for Mummy Studies in Bolzano, Italy. DNA was extracted from the tissue samples using a chloroform-based DNA extraction method—a method known to remove efficiently inhibitory substances and that has been previously already successfully applied to other mummified specimen^28,29^. Libraries for the sequencing were generated^30,31^ and 100-base pair paired-end sequencing was performed on an Illumina HiSeq2500 platform^28^. Paired Illumina reads were quality-checked and processed (adapter removal and read merging) using the SeqPrep tool. Preprocessed reads were mapped to the human genome (build Hg19^32^, default mapping parameters) using bowtie2 and the parameter “end-to-end”^32,33^. To deduplicate the mapped reads, we used the DeDup tool^34^. The minimum mapping and base quality were both 30. The resulting bam files were used to check for characteristic aDNA nucleotide misincorporation frequency patterns using mapDamage2^35^. A general taxonomic profile of the sequencing reads was assessed using DIAMOND blastx search against the NCBI nr database (Release 237, April 2020). The DIAMOND^36^ tables were converted to rma6 (blast2rma tool) format (minPercentIdentity 97), imported into MEGAN6 software^37^, and subsequently visualized using the Krona tool^38^.

### DNA methylation analysis

Genomic DNA (approximately 320 ng total DNA for the specimen of A.C.B, and 220 ng for T.H.) was bisulfite converted according to the manufacturer’s instructions and hybridized with the Illumina EPIC methylation microarray (at Life and Brain GmbH, Bonn, Germany). We did not perform additional quality control before hybridization to reduce further loss of the specimen. For comparison, we compiled 301 Illumina EPIC methylation profiles of nine different tissues from 13 different studies (Supplemental Tables S3, S4).

The IDAT files of the Illumina BeadChip datasets were normalized with ssNoob using the minfi R package. Samples with bad quality and three colon samples suspected to be mislabeled were excluded. To select of probes with reliable signal within the ancient DNA samples, we used the detection P-value with out-of-band (OOB) array hybridization (pOOBAH) approach, in the R package SeSAMe^17^. We selected all probes with a P-value < 0.01 in each mummy sample. Normalization control probe pairs, which are based on sequences of housekeeping genes (without any CpGs) are often used for normalization and processing approaches^39^. They enable to correct the dye bias between the red and green fluorescence channel, which only affects type II assay beads. Since our samples show no or just a very low signal for these probes, we focused our analysis on the type I assay probes. We also removed CpG sites associated with the X and Y chromosomes, as well as SNPs, which resulted in a subset of 22,778 CpGs for the T.H. specimen. MDS analysis was performed with the R package limma.

### Tissue deconvolution and sex determination

To estimate the tissue of origin, we initially used a previously described human cell-type methylation atlas^4^. However, only 578 out of 7890 CpGs of these signatures were detected in the T.H. specimen—therefore, only these CpGs were used for a non-negative least square (NNLS) deconvolution algorithm^40–42^ of the reference dataset and the T.H. sample. For easier comparison with our own selection, we reduced the number of groups from 25 to 10 to only represent the selected tissues in our datasets. Blood subtype predictions were combined to “Leukocytes” and the remaining groups were combined as “Other”.

Alternatively, we selected candidate CpGs for each tissue within the subset of 22,778 detected CpGs based on the difference in mean beta values and variance within the groups in the training set with a tenfold cross-validation^3^. The top 5 ranked CpGs for each of the nine tissues were then implemented into the NNLS algorithm to predict tissue proportions. The mean beta values for each tissue in the training set were used as the reference matrix.

### Training of epigenetic age predictor

For epigenetic age prediction, we used age-associated CpGs from a multi-tissue epigenetic clock^7^. Only 7 out of 353 CpGs of this signature were within our 22,778 detected CpGs. Therefore, new coefficients for a linear prediction model were calculated by fitting a multivariable linear model with the R stats package based on the training samples (Supplemental Table S5).

### Software

A table with all used software, versions and references can be found in the supplemental information (Supplemental Table S6).

### Ethics declarations

All methods were carried out in accordance with relevant guidelines and regulations as indicated above. The experiments were approved by the institute/museum where the mummies belong. This study was conducted according to the International Council of Museums code of ethics (2017) with regard to storage, display, and study of human remains.

## Supplementary Information

Supplementary Information 1.

Supplementary Information 2.

## Data Availability

Raw data of DNA methylation profiles generated in this study were submitted to Gene Expression Omnibus (GEO): GSE169595.
