# Supplementary material for: DNA methylation profiling in mummified human remains from the eighteenth-century
Source: Sci Rep. 2021 Jul 29;11:15493. doi: 10.1038/s41598-021-95021-7 (PMC8322318; doi:10.1038/s41598-021-95021-7)
Supplement: Supplementary file 1 — Supplementary Information 1. [file 41598_2021_95021_MOESM1_ESM.pdf]

Supplemental Material

DNA Methylation Profiling in Mummified Human Remains from the Eighteenth-Century

Marco Schmidt, Frank Maixner, Gerhard Hotz, Ildikó Pap, Ildikó Szikossy, György Pálfi, Albert Zink, Wolfgang Wagner

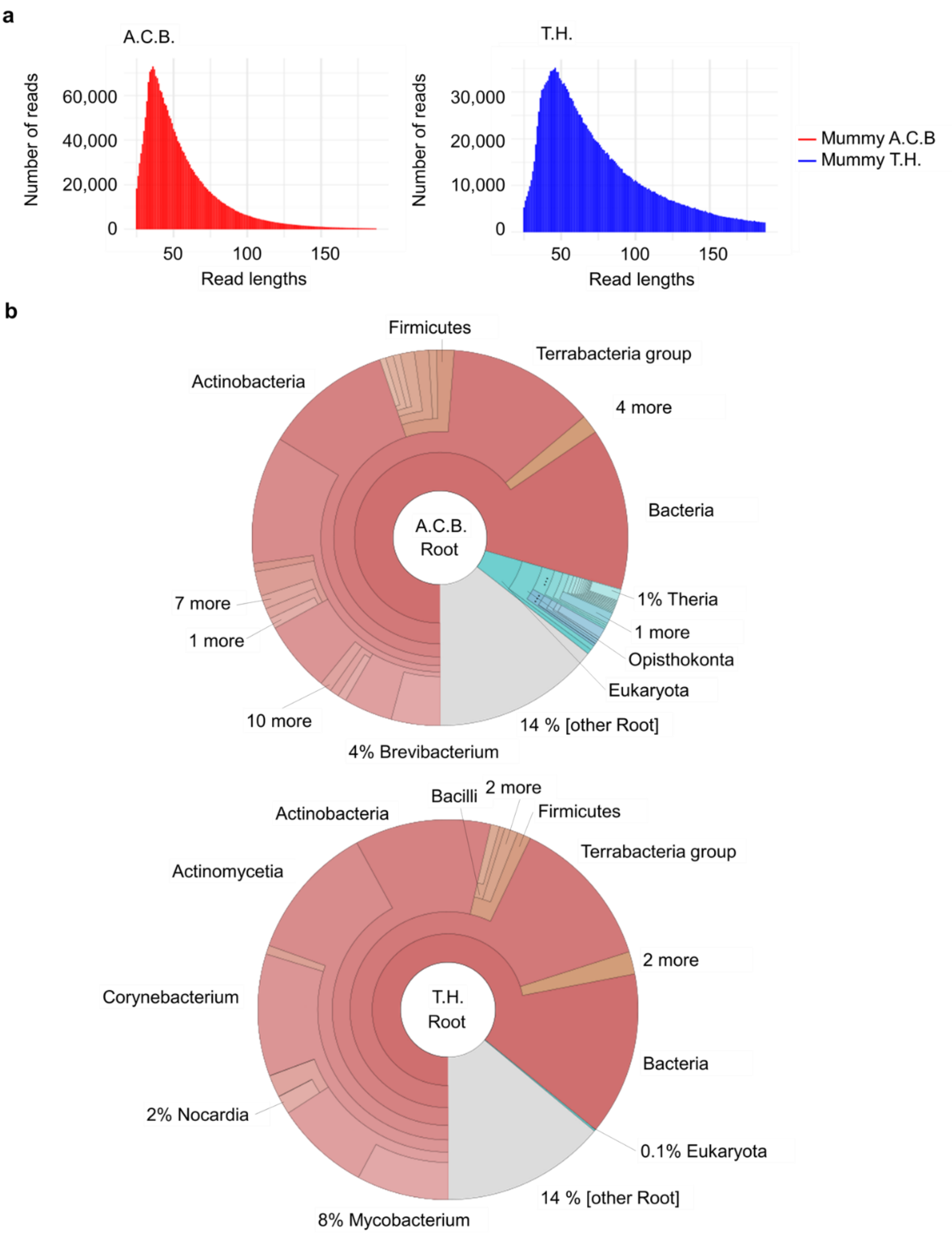

Supplemental Figure S1. Overview of the metagenomic datasets of the two mummified tissues. a) Histogram showing the read fragment length distribution in base pairs (bp). **b)** Taxonomic overview of the sequence reads in the merged shotgun datasets of the samples A.C.B. and T.H.. The metagenomic reads were taxonomically assigned using the DIAMOND tool 2.0.7 [1] against the NCBI nr database. **Software:** SeqPrep 1.2, bowtie2 1.2.1.1 [2], DeDup 0.12.8 [3].

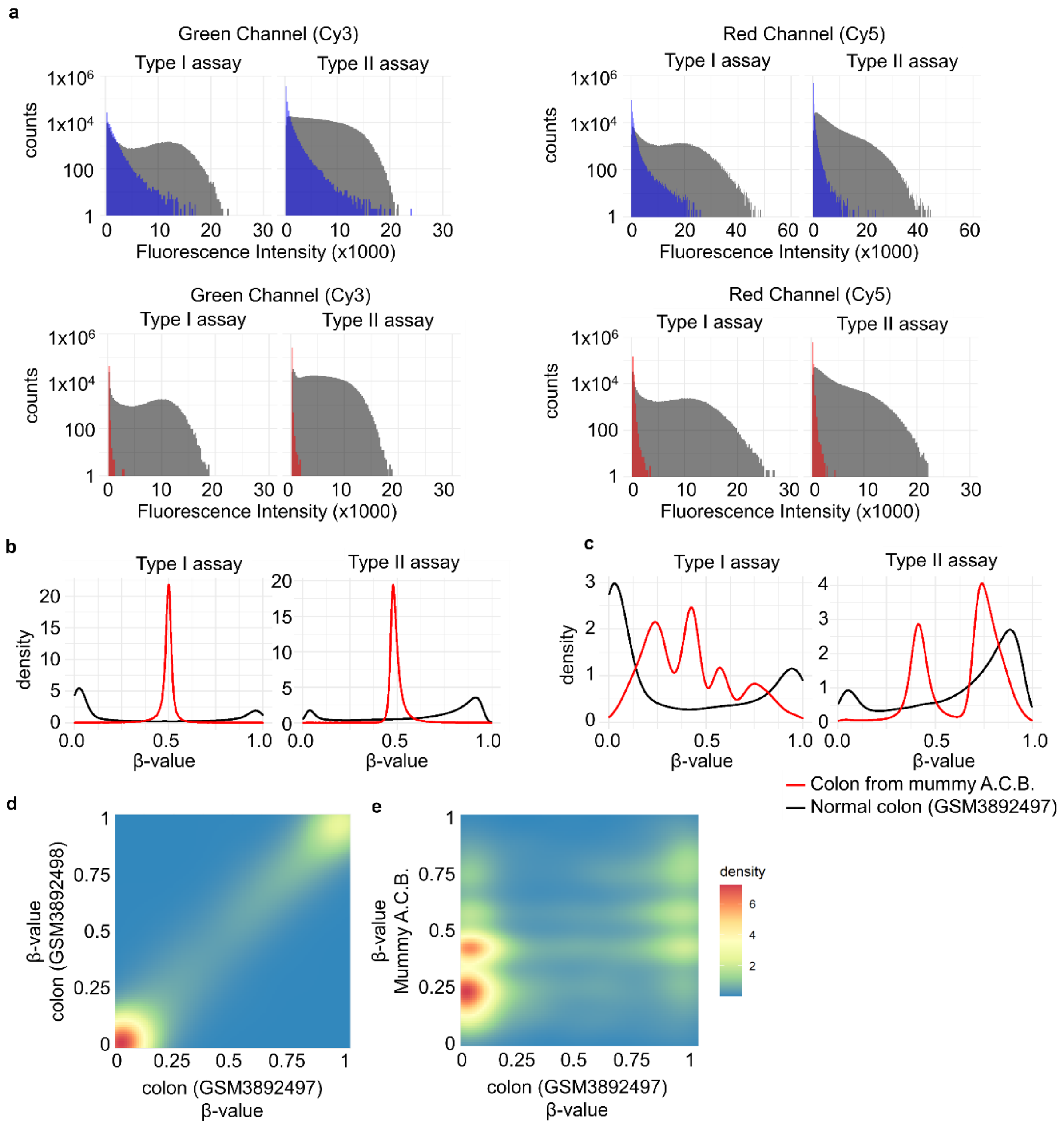

Supplemental Figure S2. Quality control of DNA methylation datasets.
a) Signal intensities of the Illumina EPIC Bead Chips for type I and type II assays in the green and red fluorescence channels. The histograms are compared for all CpGs represented on the microarray in the specimen form T.H. (blue) and A.C.B. (red) and corresponding control samples from lung tissue (GSE114989) or gut (GSE132804 and GSE149282; indicated in grey). Overall, the signal intensities were extremely low for both human remains. **b)** Density plots of DNAm levels of the A.C.B. sample (β-value) across all Type I and Type II assays represented by the Illumina EPIC BeadChip (after normalization with ssNoob). **c)** When we filtered with the SeSAMe package for CpGs that had low P-values (< 0.01) we still did not observe density plots with a typical bimodal distribution of DNAm levels. **d)** 2D density plots to compare DNAm levels in the 4,160 filtered Type I assays between two present day colon tissue samples (Pearson correlation = 0.99); and **e)** between a present-day colon tissue sample and the profile of the specimen of A.C.B. (Pearson correlation = 0.38). **Software:** R 4.0.3 [4], minfi 1.36.0 [5], ggplot2 3.3.3 [6], sesame 1.8.2. [7], reshape2 1.4.4 [8].

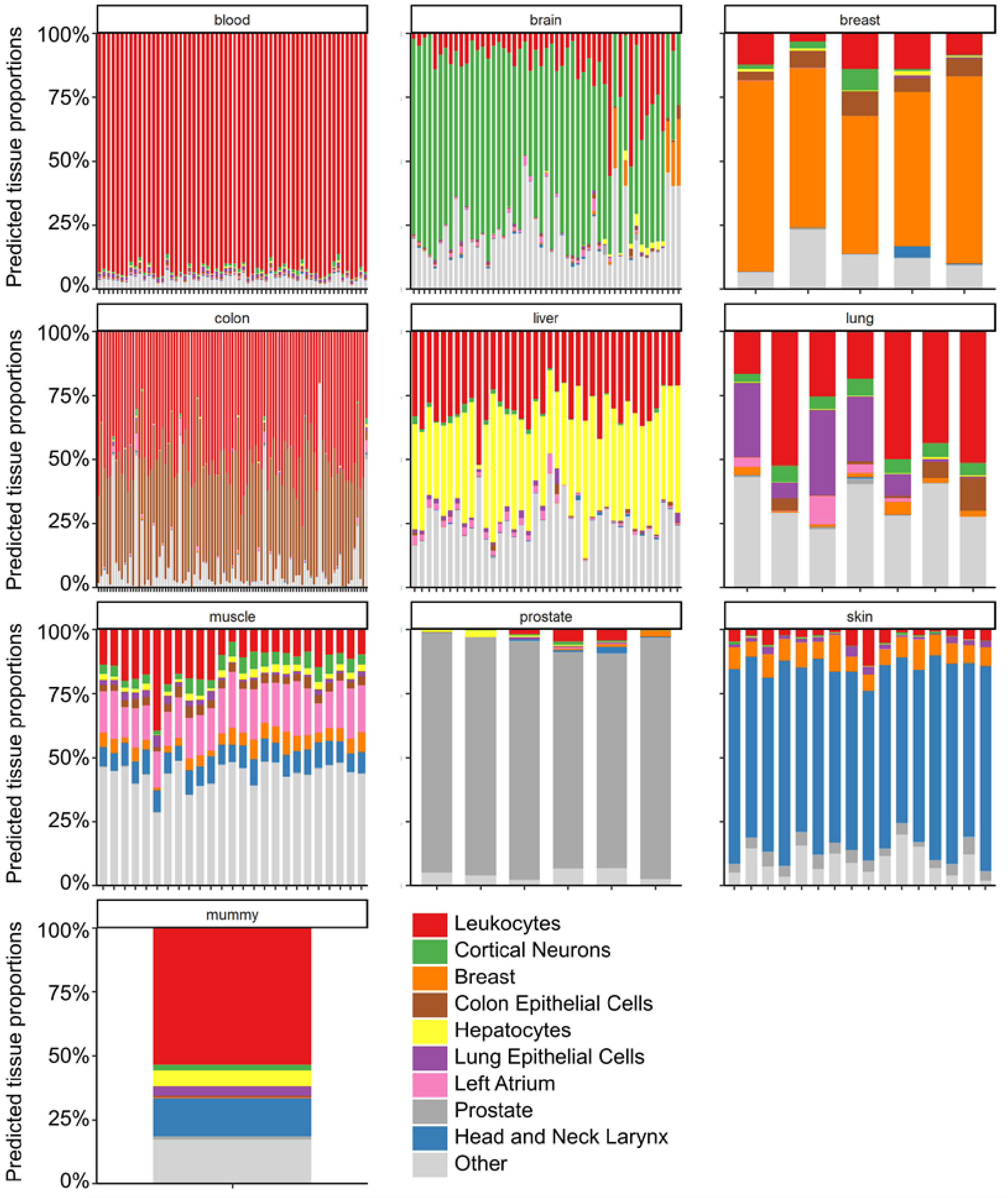

Supplemental Figure S3. Tissue-deconvolution using a reduced predictor based on Moss *et al*.. The composition of tissue was estimated for 301 public DNAm profiles and of the specimen of the T.H. mummy. This analysis was performed with a published reference methylation atlas of 25 human tissues and cell types, which utilizes 7,890 CpGs [9]. However, only 578 of these CpGs were comprised in the 22,778 CpGs that passed filter criteria for the specimen of T.H.. Despite this limitation, the predictions overall corresponded to the tissue of origin, but they were rather inconsistent for lung tissue samples and the mummy sample, which was also from lung tissue. **Software:** R 4.0.3 [4], minfi 1.36.0 [5], ggplot2 3.3.3 [6], sesame 1.8.2. [7], reshape2 1.4.4 [8].

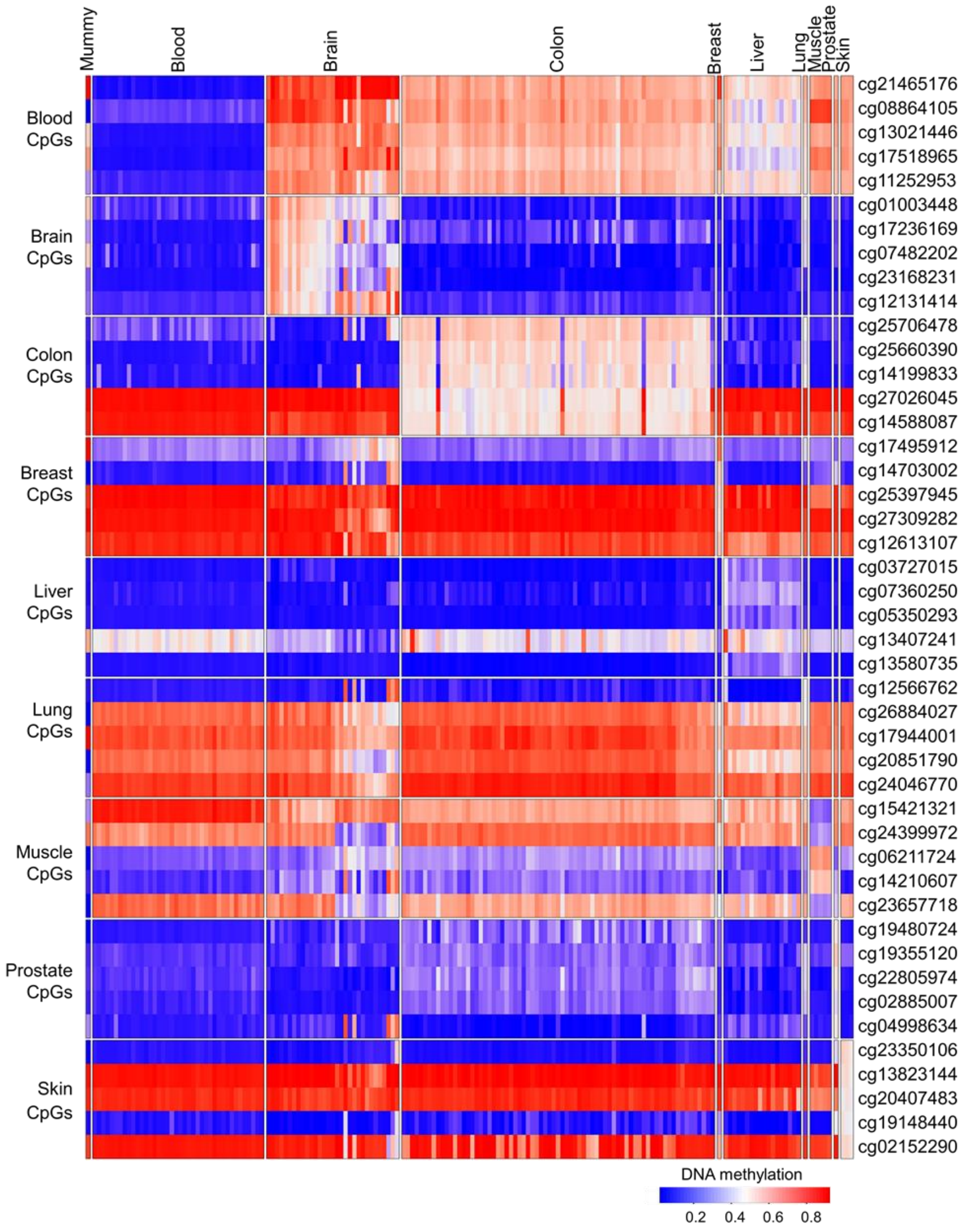

Supplemental Figure S4. Heatmap of sample-specific tissue-deconvolution matrix.
To estimate the tissue of origin in the T.H. sample, we trained a deconvolution model within the 22,778 CpGs that passed filter criteria. Our reference dataset of 301 DNAm profiles of nine different tissues was separated into a training and validation set. To find the best CpGs for tissue classification, we subjected the training data to a stratified 10-fold cross-validation based on the means and variances of DNAm values in a specific tissue and all other tissues, as described in detail before [10]. The candidates were ranked by the computed area under the precision-recall curve (AUPR) and the top 5 CpGs per tissue, which were used for deconvolution, are depicted in the heatmap for samples of the validation set. For comparison, we also depict the DNAm levels of the T.H. mummy sample on the left. **Software:** R 4.0.3 [4], minfi 1.36.0 [5], ggplot2 3.3.3 [6], sesame 1.8.2. [7], ComplexHeatmap 2.6.2 [11].

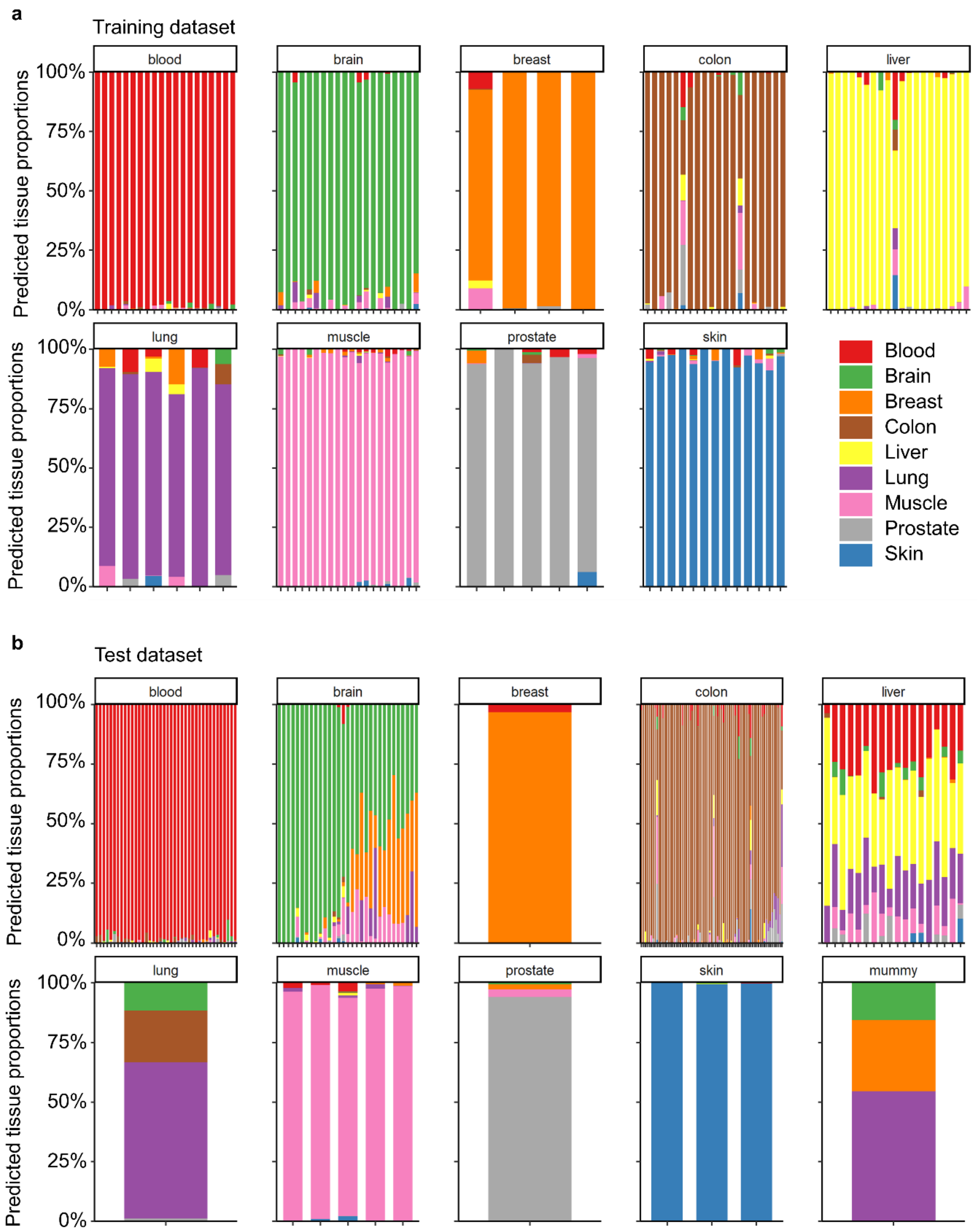

Supplemental Figure S5. Tissue deconvolution results for the training and test set. Using the cell type-specific CpGs that were selected for classification (supplemental Figure S3) a reference-based non-negative least-sequare (NNLS) algorithm was applied to estimate the composition of tissue [10]. The predicted proportions are depicted for **a)** the training set, and **b)** the validation set. Overall, predictions for the most prevalent cell types were in line with the tissue of origin. **Software:** R 4.0.3 [4], minfi 1.36.0 [5], ggplot2 3.3.3 [6], sesame 1.8.2. [7], reshape2 1.4.4 [8].

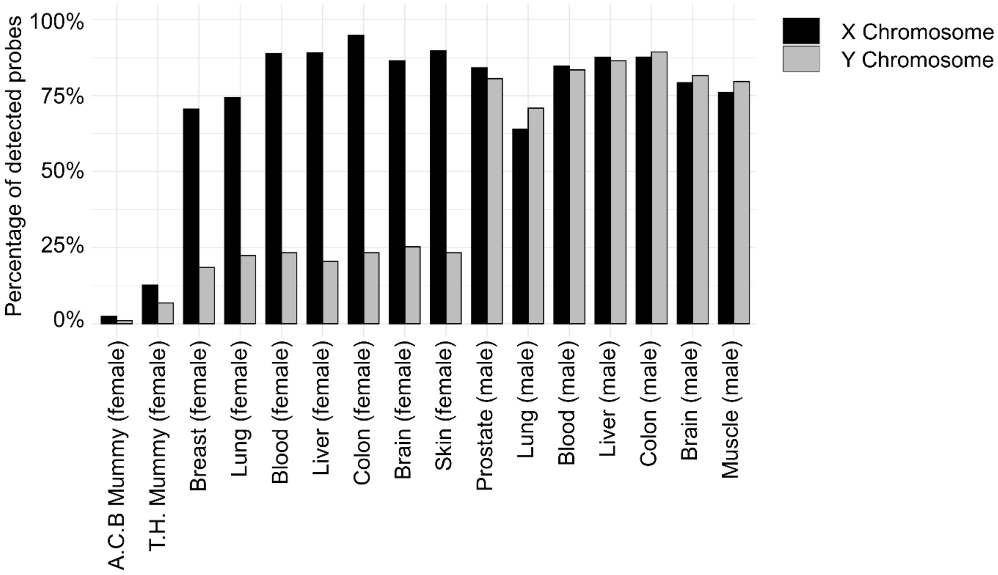

Supplemental Figure S6. Detected probes on X and Y chromosomes.

To estimate if the samples were male or female, we analyzed the percentage of type I assays probes on the X and Y chromosomes that have been filtered with detection p-values <0.01 (SeSAMe R package). Depicted are the percentages of probes (CpGs), which remain after filtering. Female samples show a lower number of detected probes for the Y chromosome. **Software:** R 4.0.3 [4], minfi 1.36.0 [5], ggplot2 3.3.3 [6], sesame 1.8.2. [7], reshape 0.8.8 [8].

Supplemental Table S1: Mapping statistics of mummies against human reference genomes.

	A.C.B.	T.H.
EURAC ID	2132	2367
Individuum	Anna Catharina Bischoff	Terézia Hausmann
Church	Bafüsserkirche	Dominican church
Site	Basel, Switzerland	Vác, Hungary
Date of death	1787	1797
Age at death (years)	68	28
Sample	gut tissue	lung tissue
Amount (mg) used for DNA extraction	100	120
DNA concentration ng/µl	16	11
No. of merged reads (SeqPrep 1.2)	25,195,825	2,081,542
No. of aligned reads to the human genome (hg19) (bowtie2 1.2.1.1, DeDup 0.12.8)	244,199	13,535
Duplication Rate	0.3	0.01
Human reads %	0.97	0.65
Mean Coverage (qualimap 2.2.1)	0.0048	0.0003
S.D	1.9744	0.0164

Supplemental Table S2: Quality control summary for Illumina EPIC BeadChip hybridizations.

Control Probes	Threshold	A.C.B.	T.H.
Restoration	0	0.0	0.0
StainingGreen	5	**1.6***	431.1
StainingRed	5	**1.0***	91.1
ExtensionGreen	5	**1.0***	99.1
ExtensionRed	5	**0.9***	24.1
HybridizationHighMedium	1	**1.0***	1.7
HybridizationMediumLow	1	1.6	2.1
TargetRemoval1	1	25.7	22.1
TargetRemoval2	1	22.1	13.9
BisulfiteConversion1Green	1	1.1	2.1
BisulfiteConversion1BackgroundGreen	1	31.6	25.0
BisulfiteConversion1Red	1	**0.9***	2.9
BisulfiteConversion1BackgroundRed	1	13.3	31.4
BisulfiteConversion2	1	1.8	2.2
BisulfiteConversion2Background	1	25.1	23.8
Specificity1Green	1	**0.8***	1.0
Specificity1Red	1	**0.7***	**0.8***
Specificity2	1	**1.0***	2.8
Specificity2Background	1	20.8	30.1
NonPolymorphicGreen	5	**1.0***	**4.1***
NonPolymorphicRed	5	**0.6***	**1.5***

*failed quality control threshold

Supplemental Table S3: Datasets used for the reference dataset.

GSE	Sample Group	Number of Samples	Reference
GSE100850	breast	5	[12]
GSE101908	prostate	6	[13]
GSE114989	lung	7	[14]
GSE147740	blood	60	[15]
GSE132399	liver	21	[16]
GSE136583	liver	17	[17]
GSE132804	colon	84	[18]
GSE149282	colon	9	[19]
GSE143157	brain	36	[20]
GSE148390	brain	15	[21]
GSE151407	muscle	25	[22]
GSE151617	skin	16	[23]

Supplemental Table S4: Samples used from the reference dataset.
Information on the 301 DNAm profiles that were used in the training and validation reference datasets. This table is provided as separate EXCEL table.

Supplemental Table S5: Epigenetic age-predictor for seven CpGs detected in the T.H. sample.

CpG Site	Gene Name	Chr	Map Info	Coefficient	P value
(Intercept)				45.81	$3.36*10^{-13}$ ***
cg23180365	GLB1, TMPPE	3	33138627	-1508.53	0.000601 ***
cg22920873	C7orf55	7	139025153	249.48	0.024648 *
cg16241714	CEBPD	8	48650511	2055.68	$5.93*10^{-6}$ ***
cg21801378	BRUNOL6	15	72612125	333.77	$5.38*10^{-5}$ ***
cg02331561	ABCA17P, ABCA3	16	2391081	248.83	$3.99*10^{-7}$ ***
cg02047577	UCKL1AS, UCKL1	20	62587702	-2470.84	0.000232 ***
cg11932564	TNFRSF13C	22	42322146	390.3	0.003193 **

*** P<0.001, ** P<0.01, * P<0.05

Supplemental Table S6: Used software and version.

Software	**Version**	**Reference**
Illumina BeadArray Controls Reporter	1.1	https://support.illumina.com/downloads/beadarray-controls-reporter-installer.html
R	4.0.3	https://www.R-project.org/ [4]
minfi	1.36.0	https://bioconductor.org/packages/release/bioc/html/minfi.html [5]
ggplot2	3.3.3	https://ggplot2.tidyverse.org [6]
ComplexHeatmap	2.6.2	https://github.com/jokergoo/ComplexHeatmap [11]
sesame	1.8.2	https://bioconductor.org/packages/release/bioc/html/sesame.html [7]
reshape	0.8.8	http://www.jstatsoft.org/v21/i12/ [8]
reshape2	1.4.4	http://www.jstatsoft.org/v21/i12/ [8]
CimpleG	0.0.1.9057	unpublished
SeqPrep	1.2	https://github.com/jstjohn/SeqPrep
bowtie2	1.2.1.1	https://github.com/BenLangmead/bowtie2 [2]
DeDup	0.12.8	https://github.com/apeltzer/DeDup [3]
mapDamage2	2.0.9	https://github.com/ginolhac/mapDamage [24]
DIAMOND	2.0.7	https://github.com/bbuchfink/diamond [1]
MEGAN6	6.18.2	https://software-ab.informatik.uni-tuebingen.de/download/megan6/welcome.html [25]
blast2rma tool		https://github.com/husonlab/megan-ce/blob/master/tools/blast2rma [25]
Krona	2.7.1	https://github.com/marbl/Krona [26]
qualimap	2.2.1	http://qualimap.conesalab.org/ [27]

Supplemental References

1. Buchfink, B., Reuter, K. & Drost, H.G. Sensitive protein alignments at tree-of-life scale using DIAMOND. *Nat Methods* **18**, 366-368 (2021).

2. Langmead, B. & Salzberg, S.L. Fast gapped-read alignment with Bowtie 2. *Nature Methods* **9**, 357 (2012).

3. Peltzer, A. *et al.* EAGER: efficient ancient genome reconstruction. *Genome Biol* **17**, 60 (2016).

4. R Core Team. R: A Language and Environment for Statistical Computing. *R Foundation for Statistical Computing* (2020).

5. Aryee, M.J. *et al.* Minfi: a flexible and comprehensive Bioconductor package for the analysis of Infinium DNA methylation microarrays. *Bioinformatics* **30**, 1363-1369 (2014).

6. Wickham, H. ggplot2: Elegant Graphics for Data Analysis. *Springer-Verlag New York* (2016).

7. Zhou, W., Triche, T.J., Jr., Laird, P.W. & Shen, H. SeSAMe: reducing artifactual detection of DNA methylation by Infinium BeadChips in genomic deletions. *Nucleic Acids Res* **46**, e123 (2018).

8. Wickham, H. Reshaping Data with the reshape Package. *Journal of Statistical Software* **21**, 1-20 (2007).

9. Moss, J. *et al.* Comprehensive human cell-type methylation atlas reveals origins of circulating cell-free DNA in health and disease. *Nat Commun* **9**, 5068 (2018).

10. Schmidt, M., Maie, T., Dahl, E., Costa, I.G. & Wagner, W. Deconvolution of cellular subsets in human tissue based on targeted DNA methylation analysis at individual CpG sites. *BMC Biol* **18**, 178 (2020).

11. Gu, Z., Eils, R. & Schlesner, M. Complex heatmaps reveal patterns and correlations in multidimensional genomic data. *Bioinformatics* **32**, 2847-9 (2016).

12. Oltra, S.S. *et al.* Methylation deregulation of miRNA promoters identifies miR124-2 as a survival biomarker in Breast Cancer in very young women. *Sci Rep* **8**, 14373 (2018).

13. Parry, M.A. *et al.* Genomic Evaluation of Multiparametric Magnetic Resonance Imaging-visible and -nonvisible Lesions in Clinically Localised Prostate Cancer. *Eur Urol Oncol* **2**, 1-11 (2019).

14. Dietz, S. *et al.* Global DNA methylation reflects spatial heterogeneity and molecular evolution of lung adenocarcinomas. *Int J Cancer* **144**, 1061-1072 (2019).

15. Robinson, O. *et al.* Determinants of accelerated metabolomic and epigenetic aging in a UK cohort. *Aging Cell* **19**, e13149 (2020).

16. Carrillo-Reixach, J. *et al.* Epigenetic footprint enables molecular risk stratification of hepatoblastoma with clinical implications. *J Hepatol* **73**, 328-341 (2020).

17. Cerapio, J.P. *et al.* Global DNA hypermethylation pattern and unique gene expression signature in liver cancer from patients with Indigenous American ancestry. *Oncotarget* **12**, 475-492 (2021).

18. Wang, T. *et al.* Dysfunctional epigenetic aging of the normal colon and colorectal cancer risk. *Clin Epigenetics* **12**, 5 (2020).

19. Ishak, M. *et al.* Genome-Wide Open Chromatin Methylome Profiles in Colorectal Cancer. *Biomolecules* **10**(2020).

20. Rydbirk, R. *et al.* Epigenetic modulation of AREL1 and increased HLA expression in brains of multiple system atrophy patients. *Acta Neuropathol Commun* **8**, 29 (2020).

21. Kanchan, R.K. *et al.* MiR-1253 exerts tumor-suppressive effects in medulloblastoma via inhibition of CDK6 and CD276 (B7-H3). *Brain Pathol* **30**, 732-745 (2020).

22. Voisin, S. *et al.* An epigenetic clock for human skeletal muscle. *J Cachexia Sarcopenia Muscle* **11**, 887-898 (2020).

23. Boroni, M. *et al.* Highly accurate skin-specific methylome analysis algorithm as a platform to screen and validate therapeutics for healthy aging. *Clin Epigenetics* **12**, 105 (2020).

24. Jónsson, H., Ginolhac, A., Schubert, M., Johnson, P. & Orlando, L. mapDamage2.0: fast approximate Bayesian estimates of ancient DNA damage parameters. *Bioinformatics* **29**, 1682-1684 (2013).

25. Huson, D.H. *et al.* MEGAN Community Edition - Interactive Exploration and Analysis of Large-Scale Microbiome Sequencing Data. *PLoS Comput Biol* **12**, e1004957 (2016).

26. Ondov, B.D., Bergman, N.H. & Phillippy, A.M. Interactive metagenomic visualization in a Web browser. *BMC Bioinformatics* **12**, 385 (2011).

27. Okonechnikov, K., Conesa, A. & Garcia-Alcalde, F. Qualimap 2: advanced multi-sample quality control for high-throughput sequencing data. *Bioinformatics* **32**, 292-4 (2016).
